# Diffuse Large B-Cell Lymphoma in Bilateral Basal Ganglia: A Rare Case Report

**DOI:** 10.7759/cureus.32743

**Published:** 2022-12-20

**Authors:** Aldo JF Da Silva, Fernando E Castro Pinheiro Gomes, Gabriele M Barros Pimentel Tenório, Lais M Pinto Almeida

**Affiliations:** 1 Pediatric Neurosurgery, Santa Mônica Teaching Maternity, Alagoas State University of Health Sciences, Maceio, BRA; 2 Pediatric Neurosurgery, General State Hospital, Maceio, BRA; 3 College of Medicine, Tiradentes University Centre, Maceio, BRA

**Keywords:** radiotherapy (rt), lymphoma, aphasia, magnetic resonance imaging, basal ganglia

## Abstract

Primary central nervous system lymphoma (PCNSL) may affect the basal nuclei of the brain. The most common type is diffuse large B-cell lymphoma. This is a case report of an adolescent with rare bilateral lymphoma in the basal ganglia. A male patient aged 16 years had a clinical picture of rapid progression to motor aphasia, dysphagia, and right hemiplegia. Magnetic resonance imaging (MRI) of the skull, along with spectroscopy, indicated lymphoproliferative or neoplastic disease. A biopsy confirmed diffuse large B-cell lymphoma. However, the patient died before starting the definitive treatment. PCNSL is infrequent in the pediatric population, but it has a better prognosis in this age group, especially when diagnosed early. It affects immunodeficient patients more often. In addition to MRI, spectroscopy and positron-emission tomography help clarify the diagnosis. Biopsy is a gold standard for diagnosis, leading to the appropriate initiation of chemotherapy and radiotherapy treatment. PCNSL is rare in young patients. With an early diagnosis, better therapeutic planning is possible. Unfortunately, in the present case, the diagnosis was late, and the patient had an unfavorable outcome.

## Introduction

Primary central nervous system lymphoma (PCNSL) is an extranodal tumor that can affect the brain, eyes, spinal cord, or leptomeninges in the absence of systemic disease. However, it commonly involves the deep periventricular white matter of the brain hemisphere, corpus callosum, and basal ganglia. It is a disease that affects approximately 1% of the population under 19 years of age [1,2].

Approximately 90% of PCNSL cases are diffuse large B-cell lymphomas, and the remaining 10% are T-cell Burkitt or indolent lymphomas [3]. These tumors can manifest as focal lesions, which may be single, multiple, or even disseminated diseases [4].

Differential diagnoses should be made between these lesions and several diseases, both systemic and focal, such as multiple sclerosis, sarcoidosis, and gliomas. Imaging and biopsy are of fundamental importance to establish appropriate treatment [3].

Herein, we report a rare case of bilateral lymphoma in the basal ganglia in an adolescent to discuss and list the key clinical and radiological findings of this condition.

## Case presentation

**Figure 1:**
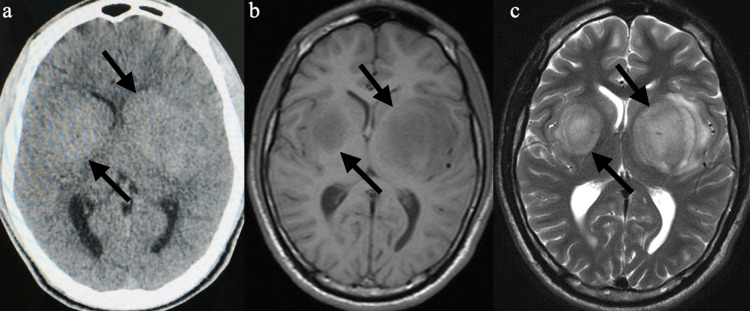
CT and MRI (a) Noncontrast computed tomography scan showing bilateral hyperattenuating lesions in the basal ganglia (black arrows); (b) Axial T1-weighted magnetic resonance image with low-intensity lesions, larger on the left, with lateral ventricle compression (black arrows); (c) Axial T2-weighted magnetic resonance image with hypersignal (black arrows)

**Figure 2:**
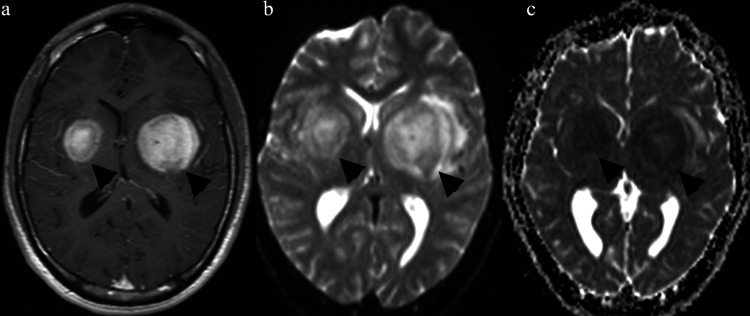
MRI (a) Axial contrast-enhanced T1-weighted magnetic resonance images showing lesions in the basal ganglia, larger on the left, with lateral ventricle compression (black arrowheads); (b) the same on the diffused-weight imaging map with signal restriction and high signal strength (black arrowheads), and (c) on the apparent diffusion coefficient map with low signal intensity (black arrowheads)

A 16-year-old male patient presenting with numbness on the right side of the body and a history of sporadic fever was treated at the emergency department. The patient denied any history of trauma or drug use and reported having been a healthy teenager until then with no previous illnesses. During hospitalization, laboratory tests were normal, and markers for infection (HIV, toxoplasmosis, syphilis) were negative. First, a non-contrast computed tomography (CT) examination of the skull was performed, which revealed a hyperdense area bilaterally in the basal ganglia (Figure 1a). Magnetic resonance imaging (MRI) of the skull revealed a bilateral homogeneous expansive process evidenced by paramagnetic contrast in the basal nuclei (larger on the left) (Figures 1b, 1c, 2a-2c). Spectroscopy sequences revealed a decrease in N-acetyl aspartate (NAA) peaks, as well as an inversion of the choline/creatinine and choline/NAA ratios and the presence of choline peaks, indicating cell proliferation.

**Figure 3:**
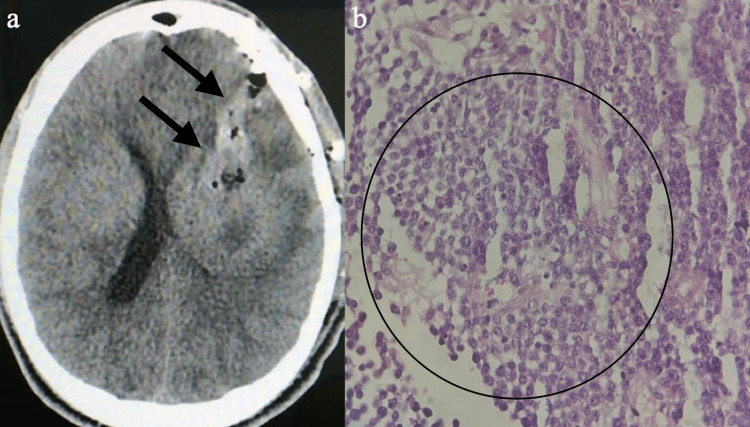
Postoperative and histopathological (a) Computed tomography axial cut, a postoperative biopsy of the left basal ganglia via craniotomy with surgical manipulation area (black arrows); (b) Round, small, blue cells with occasional mitoses (black circle), consistent with diffuse large B-cell lymphoma (H&E staining)

Thus, the etiology could be either lymphoproliferative or primary high-grade glial neoplasm. The clinical picture progressed rapidly with neurological worsening: motor aphasia, lip asymmetry, dysphagia, and right hemiplegia. In view of this situation, it was decided to perform a biopsy by craniotomy (Figure 3a). Histopathological examination (Figure 3b) showed an undifferentiated malignancy, and immunohistochemistry confirmed diffuse large-B-cell lymphoma (CD20-positive, with high Ki67 expression indicating cell proliferation, and CD20-negative for CD10, BCL6, and MUM1). However, on the sixth postoperative day, the patient’s condition worsened, with cardiorespiratory arrest and unsuccessful resuscitation.

## Discussion

PCNSL is a rare disease that represents approximately 6% of all malignant brain tumors and 1%-2% of all lymphomas [5]. The age of diagnosis is usually between the fifth and sixth decade of life. It is less frequent in pediatric patients, with a better prognosis in this age group than in the older population [1].

Acquired or congenital immunodeficiency is the best-established risk factor, and the disease usually presents as a single tumor located mainly in the frontal lobes (20%-43%) and the basal nuclei (9%-13%) [4].

The clinical picture is variable and usually presents with a rapidly progressing neurological deficit, which varies according to the location of the tumor and occasionally leads to progressive encephalopathy [1]. In the present case, since the patient had bilateral involvement of the basal nuclei, the main repercussions were on motor patterns.

Bilateral involvement of the basal nuclei is more common in immunodeficient patients, increasing the possibility of differential diagnosis. Lesions with this pattern of involvement may have a systemic or local cause, involving metabolic conditions, such as Wilson’s disease; neurodegenerative conditions, such as Fahr’s disease and vascular abnormalities; infectious diseases, such as toxoplasmosis; inflammatory conditions, such as Behçet’s disease; and neoplasms, such as primary lymphomas, gliomas, and various metastases [2].

Despite a characteristic imaging pattern in traditional MRI sequences, this method cannot differentiate PCNSL from other neoplasms or non-neoplastic diseases that affect the central nervous system. Therefore, more modern imaging modalities have been used, such as spectroscopy and positron-emission tomography (PET), which help differentiate PCNSL from other diseases in a less invasive way through their profiles of metabolic activity and biochemistry. For example, spectroscopy evidences high lipid peaks combined with high choline/creatinine ratios. PET with fluorodeoxyglucose (FDG) shows hypermetabolic lesions with increased FDG uptake [6]. In the present case, MRI showed a homogeneous bilateral lesion evidenced by paramagnetic contrast in the basal nuclei, and spectroscopy showed choline peaks.

Lesions caused by PCNSL have higher metabolic activity than metastases and high-grade gliomas. By contrast, infectious processes usually present hypometabolic lesions in immunocompromised patients, while PCNSL has a hypermetabolic pattern with high FDG rates in PET [6].

In addition to imaging tests, when there is a high diagnostic suspicion of PCNSL, a biopsy of the lesion with a histopathological study is the gold standard for the diagnosis of the disease and the establishment of an appropriate therapeutic plan [4]. Because the described case featured a bilateral lesion in eloquent areas, the diagnosis was defined only after the biopsy.

In general, surgical resection of the lesion is not indicated because PCNSL is a highly infiltrative and aggressive tumor. Even sensitive to radiotherapy and chemotherapy, the control or cure of the disease is not guaranteed. As it is an aggressive disease, it is important to develop prognostic models: 1: International Extranodal Lymphoma Study Group (IELSG) composed of five prognostic variables (age more than 60 years, Eastern Cooperative Oncology Group (ECOG) performance status more than one, elevated level of serum lactate dehydrogenase (LDH), high cerebrospinal (CSF) protein concentration, and involvement of the deep regions brainstem, and/or cerebellum); 2: Nottingham/Barcelona (NB) composed of three risk factors (age greater than or equal to 60 years, ECOG greater than one and presence of multifocal lesion); 3: Memorial Sloan-Kettering Cancer Center (MSKCC) using only two variables (age and performance status). Despite these models, there is still no consensus on the ideal [1,7].

## Conclusions

PCNSL is a rare tumor in young patients and can affect the basal nuclei of the brain bilaterally. Early diagnosis with biopsy enables better multimodal therapeutic planning and, consequently, a better response to treatment. Unfortunately, in the reported case, the diagnosis was delayed, with an unfavorable outcome for the patient.
